# Knowledge of obstetric danger signs among Saudi Arabian women

**DOI:** 10.1186/s12889-020-09075-9

**Published:** 2020-06-15

**Authors:** Amani Abu-Shaheen, Humariya Heena, Abdullah Nofal, Muhammad Riaz, Isamme AlFayyad

**Affiliations:** 1 Research Center, King Fahad Medical City, P.O. Box: 59046, Riyadh, 11525 Saudi Arabia; 2 Emergency Medicine Department, King Saud University Medical City, Riyadh, Saudi Arabia; 3 Department of Statistics, University of Malakand, Lower Dir KPK, Pakistan

**Keywords:** Danger signs, Obstetric, Postpartum, Pregnancy, Saudi Arabia

## Abstract

**Background:**

In the Middle East, tremendous efforts have been made to promote both maternal and child health. However, there is little information in the literature about maternal knowledge of obstetric danger signs. Thus, this study aims to investigate Saudi Arabian women’s knowledge of obstetric danger signs and their determinant factors.

**Methods:**

A community-based, cross-sectional survey was conducted in primary health care centers (PHCCs) in Riyadh City. A proportionate random sample of women who have delivered during the past 2 years was selected from the PHCCs. Data were then collected through structured interviews; the questionnaire was developed based on the literature review.

**Results:**

A random sample of 1397 women were included in the final analysis of this study. During pregnancy, 21.1% of the participants reported that they knew about swollen hands or faces. During labor, 23.1% of the participants reported that they knew about prolonged labor (> 12 h). At postpartum, 26.3% of the participants reported that they knew about foul-smelling vaginal discharge. In this study, women in the northern region of Riyadh who visited government hospitals had significantly higher odds of knowing at least one obstetric danger sign. A statistically significant difference was found between the participants’ knowledge of at least one obstetric danger sign and her 11–15 times of antenatal care visit.

**Conclusions:**

A dedicated health education guide, and a kingdom-wide maternal and child health preventive care promotion program could improve the health and wellbeing of expectant mothers. In these programs, it is essential to focus on birth preparedness, with danger signs recognition, and the integrate health-related data for the whole kingdom.

## Background

Maternal mortality is high worldwide; around 80% of maternal deaths are due to complications during childbirth and pregnancy periods [1, 2]. Although most maternal deaths are preventable [3], the average maternal mortality rate is 216 per 100,000 live births in developing countries. In contrast, in developed countries, it is 12 per 100,000 live births [1, 2].

Obstetric danger signs are simply symptoms that well known to non-medical people. The most prevalent danger signs occurring during pregnancy are severe vaginal bleeding, swollen hands or face, and blurred vision. During labor, the most common danger signs are severe vaginal bleeding, prolonged labor, convulsions, and retained placenta. Conversely, severe bleeding, loss of consciousness, and fever are the most frequent danger signs occurring during postpartum [4, 5]. Women should learn to recognize these danger signs and their association with obstetric complications so they and their families could seek prompt medical care, thus ensuring safe delivery for both the mother and her child [6]. Lack of awareness of obstetric danger signs contributes to delays in receiving essential obstetric care. Increased maternal knowledge of obstetric danger signs is crucial for decreasing the delay in seeking obstetric care and promoting maternal and child health [7, 8].

In Saudi Arabia, the maternal mortality rate is relatively low, with only seven deaths for every 100,000 live births [9]. This may be due to free, easy access to skilled care at birth and emergency obstetric care for other complications. Furthermore, the cultural preferences that promote facility delivery have also contributed to the low mortality rate. Such is not the case in many other countries. However, the Saudi population is unique in that there is a strong preference for marriages between cousins in the general population [10]. Moreover, Saudi Arabians also prefer marrying at relatively early ages. These circumstances would likely increase the danger of pregnancy among Saudi Arabian women.

Moreover, being prepared for birth by recognizing danger signs and symptoms may empower the women and their families to make prompt decisions in dire situations. However, there is little information in the literature about maternal knowledge of obstetric danger signs. Thus, this study aims to investigate Saudi women’s knowledge of obstetric danger signs and their determinant factors.

## Methods

A community-based, cross-sectional survey was conducted in primary health care centers (PHCCs) of the Ministry of Health (MOH) in all districts of Riyadh City, Saudi Arabia. Riyadh is the largest city in Saudi Arabia, with a population of 4.21 million people. According to data from 2017 to 2018, more than 30% of the Saudi Arabian population are expatriates [11].

Around 10% of the PHCCs in each district were selected randomly. A proportionate random sample of women who have delivered during the past 2 years was then selected from the PHCCs. Women who were physically or mentally unable to provide information were excluded from this study.

Data were then collected through a structured interview with the participants in order to include those who were illiterate. The questionnaire was developed based on a literature review [1–8] and piloted to resolve any difficulties or ambiguities.

The research assistants collected data about the participants’ socio-demographic characteristics, including age, area of residence, nationality, education level, employment, place of antenatal care (ANC), postnatal care (PNC) visits, monthly household income, number of children (defined by the number of live births), and their responses to questions about their reproductive history and morbidities. They were asked to list any of the obstetric danger signs that may have occurred during the antenatal period, delivery, and postpartum. The key danger signs during the antenatal period include: severe vaginal bleeding, swollen hands or face, and blurred vision. On the other hand, key danger signs during delivery are: severe vaginal bleeding, prolonged labor (labor lasting for more than 12 h), convulsions, and retained placenta. The key danger signs during postpartum include: severe vaginal bleeding, foul-smelling vaginal discharge, and high temperature. The participants were asked: “what are the serious health problems that might happen to a woman during pregnancy, labor, and in the first two days after birth that could endanger her life?” Such a question was asked to elicit responses on their knowledge of key obstetric danger signs during the three stages of pregnancy. Only spontaneous responses from the participants were recorded.

Written informed consent was obtained from each participant. The Directorate of PHCCs, MOH approved the study, and ethical approval was obtained from King Fahad Medical City, Riyadh, Saudi Arabia.

Using the 2.6% fertility rate and the 200,000 estimated population of neonates at risk across Saudi Arabia, the estimated sample size (*n* = 1425) for the study was calculated. This has assumptions of precision = 1.00%, prevalence = 1.25%, population size = 200,000 and 95% Confidence interval specified limits of 0.25–2.25% (limits are equal to prevalence plus or minus precision).

The data were initially recorded with Microsoft Excel (version 13) and statistically analyzed on Stata (version 12). Frequencies (percentages) were used to describe socio-demographic characteristics, reproductive history, ANC, PNC, comorbidities, and treatment received. Frequencies (percentages) had a 95% confidence interval.

## Results

A random sample of 1397 women was interviewed and included in the final analysis. Seventy percent of the participants were 31–40 years of age; 83% were unemployed, 73.1% were Saudi Arabians, and 39% had very low income. Around 38% had four or more children.

### Participants’ knowledge of obstetric danger signs

Table 1 presents the women’s knowledge of obstetric danger signs during pregnancy (antenatal, at delivery, and postnatal periods). During pregnancy, 21.1% of the participants reported that they knew about swollen hands or faces, 15.3% reported severe vaginal bleeding, and 13.9% reported blurred vision. Seventy percent of the participants knew at least one of these three danger signs during the antenatal period. The proportion of women who knew about severe vaginal bleeding was considerably lower, from 15.3% during the antennal period to only 5.4% during labor and 9.5% during the postnatal period (*p* < 0.001). From the sample, 23.1% of the women reported knowing about prolonged labor (> 12 h), 15.5% knew about the retention of placenta, and 6.5% knew about convulsions during labor. At postpartum, 26.3% of the participants reported that knew about foul-smelling vaginal discharge, while 11.9% reported knowledge of high fever. Conversely, 38% reported knowing at least one of the signs.

**Table 1:** Participants’ knowledge of obstetric danger signs (*n* = 1397)

Knowledge of Danger Signs:		
During Pregnancy	***n*** (%)	95% CI
Severe vaginal bleeding	417 (29.9)	27.5–32.3
Swollen hands/face	628(45.0)	42.4–47.7
Blurred vision	552 (39.5)	36.9–42.1
Knowledge of at least one of the above	974 (69.7)	67.2–72.1
Knowledge of all the three above	189 (13.5)	11.8–15.4
**Intrapartum period**		
Severe vaginal bleeding	128 (9.2)	7.8–10.9
Prolonged labor (> 12 h)	522 (37.4)	34.8–40.0
Convulsions	336 (24.1)	21.9–26.5
Retained placenta	496 (35.3)	32.8–37.9
Knowledge of at least one of the above	693 (49.6)	47.0–52.2
Knowledge of all the three above	47 (3.4)	2.4–4.3
**Postpartum**		
Severe vaginal bleeding	171 (12.2)	10.5–14.0
Foul-smelling vaginal discharge	511 (36.6)	34.0–39.2
High fever	384 (27.5)	25.2–29.9
Knowledge of at least one of the above	535 (38.3)	35.7–40.9
Knowledge of all the three above	47 (3.4)	2.5–4.4

The study results indicated that women in the northern region of Riyadh OR = 1.51 (95% CI, 1.00–2.28) who visited government hospitals OR = 1.76 (1.06–2.93) had significantly higher odds of knowing at least one obstetric danger sign. Conversely, being a Saudi Arabian OR = 0.71 (0.54–0.93) and being unemployed OR = 0.53 (0.36–0.79) resulted in significantly lower odds of knowing about obstetric danger signs (Table 2).

**Table 2:** Participants’ characteristics and their association with knowledge of at least one obstetric danger signs (*N* = 1397)

Variables	Descriptive statistics	Knowledge of at least one obstetric danger signs
**Participants’ Age (in years)**	n (%)	OR (95% CI)
18–25	309 (22.1)	1 (reference)
26–30	402 (28.8)	1.00 (0.72–1.37)
31–40	583 (41.7)	1.07 (0 .79–1.44)
> 40	102 (7.3)	0.93 (0. 57–1.50)
New-born baby age (in months)^*^		
1–6	575 (42.1)	1
7–12	483 (34.6)	0.98 (0.76–1.28)
13–18	141 (10.1)	1.17 (0.77–1.76)
19–24	187 (13.4)	0.85 (0.60–1.20)
**Riyadh region**		
Central	234 (16.8)	1
West	234 (16.8)	0.94 (0.64–1.39)
East	510 (36.5)	0.90 (0.65–1.26)
South	186 (13.3)	1.06 (0.70–1.61)
North	233 (16.7)	**1.51 (1.00–2.28)**
**Participants’ nationality (Saudi)**	1044 (73.1)	0.71 (0.54–0.93)
**Level of education**		
Illiterate	87 (6.2)	1
Primary school	87 (6.2)	1.04 (0.59–1.83)
Middle school	183 (13.1)	0.89 (0.56–1.40)
Secondary school	522 (37.4)	1.13 (0.76–1.67)
Bachelor	421 (30.1)	1.43 (0.95–2.15)
Higher education	38 (2.7)	1.23 (0.56–2.68)
**Employment**		
Government	170 (12.2)	1
Private sector	63 (4.5)	0.67 (0.35–1.32)
Unemployed	1164 (83.3)	**0.53 (0.36–0.79)**
Place of antenatal and postnatal care visits^*^		
Primary center	239 (17.1)	1
No care	136 (9.7)	1.10 (0.69–1.74)
Private hospital	131 (9.4)	1.54 (0.94–2.51)
Governmental hospital	126 (9.0)	**1.76 (1.06–2.93)**
Household monthly income (Saudi Riyals)^*^		
0–5000	542 (38.8)	1
6000–10,000	426 (30.5)	1.29 (0.97–1.71)
10,000 and above	132 (9.5)	1.20 (0.79–1.85)
Mean number of children-parity (SD)^*^	3.1 (1.9)	1.01 (0.95–1.08)
Mean number of pregnancies (Gravidity)^*^	3.5 (2.0)	1.00 (0.95–1.06)
**Mean number of abortions**^**†**^	0.8 (1.0)	1.01 (0.86–1.19)
**Mean number of still births**^**†**^	0.1 (0.3)	1.08 (0.57–2.03)
**Any health problem during pregnancy**	378 (27.1)	**1.61 (1.27–2.06)**

^*^Data is missing in income for (*n* = 297) participants, newborn baby’s age 11, parity 15, and number of pregnancies 10 places of care 765

^†^ Number of abortions was reported by 672 and the number of stillbirths was reported by 481

A statistically significant difference was found between a participant’s knowledge of at least one obstetric danger sign and her ANC visit. It was found that 11–15 visits had better outcomes than 0–5 antenatal visits. (Table 3).

**Table 3:** Association of antenatal and postnatal care visits with knowledge of at least one obstetric danger signs (*n* = 1397)

Variables	Descriptive Statistics a	knowledge of at least one obstetric danger signs, OR (95% CI)b
Number of antenatal care visits		
0–5	169 (12.1)	1
6–10	639 (45.7)	1.36 (0.96, 1.94)
11–15	413 (29.6)	**1.68 (1.15, 2.46)**
≥ 16	152 (10.9)	1.37 (0.86, 2.18)
Number of postnatal care visits		
0–5	1151 (94.5)	1
6–10	65 (5.4)	1.47 (0 .81, 2.65)

“Number of antenatal visits” is missing for (*n* = 24) participants. A number of postnatal care visits is missing for 366, and one outlier were excluded from the analysis.

a Frequencies, percentages, mean and standard deviation.

b Odds Ratios, (95% Confidence Interval).

## Discussion

The birth of a baby is celebrated as a harbinger of hope and happiness. The role of women in bringing this happiness to families cannot be underestimated. However, women often do not get the respect they deserve, especially as mothers.

The World Health Organization (WHO) has recognized the “three delays” that contribute most to maternal mortalities across countries: delay in deciding to seek care, delay in reaching a place of care, and delay in receiving appropriate and adequate care [12]. Women must be aware of the danger signs of obstetric complications that occur during pregnancy as well as the intrapartum and postpartum periods.

Our study found that the women had very low knowledge of the danger signs to look for during pregnancy, the intrapartum, or the postpartum period. This finding is consistent with a study conducted by Krishna et al., which found that women had very little knowledge of the danger signs [13].

In this study, higher odds of knowing at least one obstetric danger sign during pregnancy were significantly associated with the region of Riyadh, which was the place of delivery. Conversely, lower odds of the knowledge were significantly associated with being a Saudi national and being unemployed. This might be because most women treated in the PHCCs were Saudi Arabians. Our study found no association between the knowledge of danger signs and the mother’s education or age, contrary to what was found by another similar study [14].

In the present study, severe vaginal bleeding as a danger sign was less recognized by the participants of the study than swollen hands or faces and blurred vision. On the other hand, documented data in other studies show that more women were aware of vaginal bleeding. Moreover, knowledge for other danger signs was found to be similar, if not lower. A study in Madagascar reported that its participants mentioned key danger signs such as fever, headache, swollen hands and body, and vaginal bleeding; the last sign somehow correlated with our study [15].

In a study conducted by Nithya et al., severe abdominal pain was recognized by 56.5% of the women in their sample to be the most common danger sign during pregnancy, followed by heavy bleeding [16]. This can be attributed to the lack of both awareness programs at the primary care level and community awareness through social media platforms. Acharya et al. and Mukhopadhyay et al. documented that only 27.8 and 37.2% of the women in their samples knew any one danger sign during pregnancy, respectively [17, 18]. In contrast, 69.7% of women in our study knew at least one danger sign. This is a healthy indication of the awareness level among Saudi Arabian women compared to their south Asian counterparts [17, 18]. Regarding the danger signs during labor, our study showed that a higher percentage of women knew prolonged labor was a danger sign.

Bhumi et al. found that the danger signs recognized by women during the postnatal period were severe bleeding (74.0%), foul-smelling discharge (72.6%), and high fever (68.6%) [19]. Conversely, our study found a higher percentage recognized foul-smelling vaginal discharge as a danger sign, while a lower percentage knew that high fever is a danger sign during the postpartum period. This is because there is no question that educated women are more informed and can take care of themselves better. Education provides better health knowledge, improves the effectiveness of health behavior, and enables women to take prompt measures when danger signs arise.

In our study, women recognized more danger signs that might occur during pregnancy, followed by the intrapartum and postpartum periods. Our finding is in agreement with a study conducted in Ethiopia, where the participants were most knowledgeable about danger signs during their pregnancy period [20]. In contrast to our findings, a study conducted in Uganda found that the participants’ knowledge of at least one danger sign was highest during childbirth and the postpartum period [8]. Moreover, in a Tanzanian study, knowledge of at least one danger sign was highest during postpartum [21]. ANC is available in Saudi Arabia, with women having uncomplicated pregnancies offered almost 12 appointments throughout their pregnancy, starting in their first trimester. In our study a statistically significant difference was found between a participant’s knowledge of at least one obstetric danger sign and her ANC visit. It was found that 11–15 visits had better outcomes than 0–5 antenatal visits. These results are similar to other study reiterating the fact that women who attend ANC are more likely to know obstetric danger signs during both pregnancy and delivery [21].

It is important to note that, since the data in our study was self-reported, there may be interviewer biases and some other biases. Our study included women who were more than 2 years past the date of their child’s delivery.

## Conclusions

A dedicated health education guide and a kingdom-wide maternal and child health preventive care promotion program could improve the health and wellbeing of expectant mothers. In creating these programs, it is essential to focus on birth preparedness with danger signs recognition and the integration of the health-related data for the whole kingdom.

## Data Availability

Data are available with the corresponding author.

## Abbreviations

ANC: Antenatal Care
CI: Confidence Interval
MOH: Ministry of Health
OR: Odds Ratios
PNC: Postnatal Care
PHCCs: Primary Health Care Centers
